# Vinblastine chemotherapy in adult patients with langerhans cell histiocytosis: a multicenter retrospective study

**DOI:** 10.1186/s13023-017-0651-z

**Published:** 2017-05-22

**Authors:** Abdellatif Tazi, Gwenaël Lorillon, Julien Haroche, Antoine Neel, Stéphane Dominique, Achille Aouba, Jean-David Bouaziz, Constance de Margerie-Melon, Emmanuelle Bugnet, Vincent Cottin, Thibault Comont, Christian Lavigne, Jean-Emmanuel Kahn, Jean Donadieu, Sylvie Chevret

**Affiliations:** 1 Assistance Publique-Hôpitaux de Paris, Hôpital Saint-Louis, Centre National de Référence de l’Histiocytose Langerhansienne, Service de Pneumologie, 1 Avenue Claude Vellefaux, 75475 Paris Cedex 10, France; 2 Université Paris Diderot, Sorbonne Paris Cité, Inserm UMR-1153 (CRESS), Biostatistics and Clinical Epidemiology research team (ECSTRA), Paris, France; 3 Assistance Publique-Hôpitaux de Paris, Hôpital Pitié Salpêtrière, Service de Médecine Interne, Université Pierre et Marie Curie, Paris, France; 4 CHU de Nantes, Hôpital Hôtel Dieu, Service de Médecine Interne, Nantes, France; 5 Département de Pneumologie, Hôpital Charles Nicolle, Rouen, France; 6 Assistance Publique-Hôpitaux de Paris, Hôpital Cochin, Service de Médecine Interne, Paris, France; 7 Assistance Publique-Hôpitaux de Paris, Hôpital Saint-Louis, Département de Dermatologie, Univ Paris Diderot, Sorbonne Paris Cité, Inserm U976, Paris, France; 8 Assistance Publique-Hôpitaux de Paris, Hôpital Saint-Louis, Service de Radiologie, Paris, France; 9 Groupement Hospitalier Est, Service de Pneumologie, Université Claude Bernard Lyon 1, Lyon, France; 10 Institut Universitaire du Cancer de Toulouse-Oncopôle, CHU de Toulouse, Service de Médecine Interne, Toulouse, France; 11 CHU Angers, Service de Médecine Interne et vasculaire, Angers, France; 12 Université Saint-Quentin en Yvelines, Hôpital Foch, Service de Médecine Interne, Suresnes, France; 13 Assistance Publique-Hôpitaux de Paris, Hôpital Trousseau, Centre National de Référence de l’Histiocytose Langerhansienne, Service d’Hémato-Oncologie pédiatrique, Paris, France; 14 Assistance Publique-Hôpitaux de Paris, Hôpital Saint-Louis, Service de Biostatistique et Information Médicale, Paris, France

**Keywords:** Langerhans cell histiocytosis, Vinblastine, Recurrence, Lung, Toxicity

## Abstract

**Background:**

Vinblastine is the standard treatment for children with Langerhans cell histiocytosis (LCH). Whether this treatment could be extended to adults with LCH is questionable. This retrospective multicenter study included 35 adult patients (median age 33 years; 23 men; 80% with multisystem LCH) who were treated with vinblastine + steroids as a first-line chemotherapy and followed for a median time of 83 months. The objectives were to determine the overall response rate (based on the Histiocyte Society criteria), disease reactivation rate, toxicity, permanent consequences, and survival rate corresponding to this treatment. The lung involvement outcome was based on serial lung function tests. The distribution of right-censored end points was estimated by the Kaplan-Meier method. Univariate Cox model with time-fixed and time-varying covariates was used for the predictive analysis of reactivation in the responders. Univariate analyses of risk factors for neurotoxicity were based on nonparametric Wilcoxon rank sum tests and exact Fisher tests.

**Results:**

The median duration of the first course of vinblastine was 7.6 months, with a median cumulative dose of 160 mg [IQR 120–212]. Seventy percent of the patients were responders at the end of this treatment. Subsequently, LCH reactivation occurred with a 5-year cumulative incidence of 40%. During the study, 27 reactivations were observed in 17 patients, and half of these episodes were retreated with vinblastine. At the end of the last vinblastine treatment, 70% of the patients were responders. None of the patients with impaired lung function improved. No grade 3–4 peripheral neuropathy was observed. At the final vinblastine treatment, permanent LCH consequences, primarily pituitary stalk involvement, were present in 15 (43%) patients, and all were present at the time of vinblastine initiation. The 10-year survival rate was 86.2% (95CI, 71.8–100%), and the 2 patients who died from LCH had risk organ localizations.

**Conclusions:**

Vinblastine is an effective and well-tolerated first-line treatment for adult LCH except in patients with lung involvement and impaired lung function. However, a significant portion of patients experienced LCH reactivation during long-term follow up. As in childhood LCH, the presence of risk organ involvement has a negative impact on patient prognosis.

## Background

Langerhans cell histiocytosis (LCH) is a rare disease of unknown etiology that can manifest in patients of all ages from neonates to the elderly, with the peak incidence occurring in adults between 20 and 40 years of age [1–4].

The clinical presentation and prognosis of LCH are highly variable. The Histiocyte Society (HS) classifies the clinical forms of LCH according to the number and type of organs involved [5]. Single-system (SS) LCH affects only one organ/system, particularly bones (unifocal or multifocal) and lungs in adults [3, 4, 6, 7]. Multisystem (MS) LCH involves two or more organs/systems, and certain localizations are known as “risk organs” (RO), including the hematopoietic system, spleen, and liver, because they present less favorable prognoses [3, 4].

LCH treatment depends on the site and extent of the disease [8, 9]. Careful observation and local treatment are usually the first steps for managing SS LCH. Patients with unifocal bone disease (UFB) may be cured by bone curettage or intralesional injection of steroids [8, 9]. Similarly, in patients with isolated mild pulmonary LCH, smoking cessation is frequently the only therapeutic intervention [6, 7, 10]. However, systemic therapy is indicated in patients with MS LCH as well as in a proportion of patients presenting bone involvement and local extension to soft tissues that may induce neurological dysfunction (referred as “risk” bone lesions) and in certain patients suffering from multifocal bone disease (MFB) [8, 9].

Unlike pediatric LCH treatment, a standard first-line chemotherapy treatment is not available for adult LCH patients [8, 11–14]. Although vinblastine + steroids is the standard treatment of LCH [15–17], the efficacy and tolerance of this treatment in adult patients are not well understood. A monocentric retrospective study comparing different chemotherapy regimens for adult bone LCH lesions reported that the combination of vinblastine + steroids was inefficient and associated with a high rate (75%) of grade 3–4 adverse events that necessitated treatment cessation [11].

These negative results from the use of vinblastine + steroids in adult LCH patients do not correspond to anecdotal clinical practice experience. Of note, the LCH-III protocol designed for RO-negative pediatric LCH was also recommended for adult patients [17].

To address this important issue, we conducted a multicenter retrospective study on adult LCH patients who were treated with vinblastine, and the objectives were as follows: 1) describe the modalities of the use of vinblastine + steroids in adult LCH patients; and 2) determine the overall response rate, disease reactivation rate, toxicity, permanent consequences, and survival rate corresponding to this treatment.

## Methods

### Study design and subject selection

This retrospective study was conducted by the French National Reference Center for LCH in collaboration with 8 teaching hospital departments. Patients 18 years of age or older who were treated with vinblastine + steroids between 1995 and 2009 were eligible for the study.

Adult patients who were previously treated with another type of chemotherapy were excluded. However, patients who received chemotherapy several years prior for childhood LCH and who were subsequently treated with concomitant vinblastine and steroids in adulthood were eligible for this study. The last patient follow up was June 2014.

The diagnosis of LCH was either histologically confirmed by a biopsy of an involved site or was based on a typical lung high-resolution computed tomography (HRCT) pattern eventually associated with a typical LCH localization (i.e., lytic bone lesion, diabetes insipidus, sclerosing cholangitis) and the exclusion of alternative diagnoses [7].

The study was performed in accordance with the Helsinki Declaration and approved by the Institutional Review Board (CPP Ile de France IV, IRB number 00003835). All patients provided written informed consent for the use of their medical reports for research.

### Data collection

Data on the patient demographics, smoking habits, clinical symptoms, clinical signs, and LCH localizations at the time of vinblastine initiation and during follow up were retrieved from the medical records. Bone imaging and lung CT scans were interpreted by an experienced radiologist (C de MM). Stratification of LCH was performed according to the HS criteria [5].

Because of the potential neurological toxicity of vinblastine, the following neuropathological risk factors were also recorded: alcohol abuse, diabetes, previous neurological disorders, and previous use of other medications known to cause neurological toxicity (particularly thalidomide for skin involvement).

### Evaluation of the disease state and response to treatment

The disease state was assessed based on standard evaluations as defined by the HS criteria [18]. If all signs and symptoms were resolved, the patients were considered as having non-active disease (NAD). Otherwise, they were classified as having active disease (AD). AD was further subdivided into regressive (improvement of symptoms or signs, with no new lesions), stable (persistence of symptoms or signs, with no new lesions) or progressive (progression and/or appearance of new lesions) disease.

The response of LCH patients to the first course of treatment was categorized as follows: 1) responders with either complete resolution (NAD) or regression (AD/better), 2) intermediate responders (AD stable or mixed, i.e., new lesions in one site and regression in another site), or 3) non-responders (progression) [18]. For bone lesions, regression or stability were considered response variables [15].

Because specific LCH treatments have virtually no influence on pituitary involvement, the pituitary was not considered in LCH staging unless a new endocrine dysfunction occurred (progressive disease) [8].

The outcome of pulmonary LCH involvement was based on serial lung function tests [19]. Additionally, the occurrence of a new pneumothorax during follow up was considered a sign of pulmonary LCH progression [19].

The overall response was defined as the cumulative number of patients with either NAD or AD/better at the last time of vinblastine discontinuation.

Reactivation was defined as the occurrence of a new LCH localization after the previous course of vinblastine treatment in the responsive patients. Among the intermediate responders, worsening after treatment was considered reactivation. The organs involved, the time to reactivation/worsening and the treatments used for LCH recurrence were recorded. Lung involvement reactivation/worsening was defined by either the occurrence of new pneumothorax or deterioration of lung function parameters [19].

Permanent consequences were also recorded at the time of initiation and at the last time of vinblastine discontinuation [20].

Patient status (alive vs. deceased) at the time of last follow up determined the overall survival.

### Toxicity

Neurological and other types of toxicities of both steroids and vinblastine were recorded and graded according to the National Cancer Institute (NCI) Common Terminology Criteria for Adverse Events (CTCAE).

### Endpoints

The primary outcome was a response after the first course of vinblastine + steroids as defined above.

Secondary outcomes included the occurrence of reactivation/worsening of LCH during the follow-up period, the overall and specific involvement of lung LCH responses to treatment, and the occurrence of side effects; specifically, neurological toxicity, permanent consequences and survival.

### Statistical analysis

Summary statistics that included the median with interquartile range [IQR] or percentages were calculated. The distribution of right-censored end points (time to reactivation or worsening, overall survival from date of first treatment course) was estimated by the Kaplan-Meier method. The time to relapse in responders was similarly estimated from the date of response to the date of disease recurrence or last follow up.

Univariate Cox model with time-fixed (baseline characteristics) and time-varying (treatment duration) covariates was used for the predictive analysis of reactivation in the responders. Univariate analyses of risk factors for neurotoxicity were based on nonparametric Wilcoxon rank sum tests and exact Fisher tests.

Statistical analyses were performed using SAS (SAS Inc, Cary, NC, USA) and R (https://www.R-project.org/) software. All tests were two-sided, with *p*-values of 0.05 denoting statistical significance.

## Results

### Study population

The medical records of 41 patients were identified as eligible for the study. Six patients were secondarily excluded: one patient has mixed LCH and Erdheim-Chester disease (ECD); one patient had received methotrexate and etoposide one year before vinblastine; and 4 patients did not have available medical records.

The characteristics of the remaining 35 patients (median [IQR] age: 33 [28–42] years; 23 men, all Caucasians) at the time of initiation of vinblastine are detailed in Table 1. The diagnosis of LCH was histologically confirmed in 34 patients. The remaining patient had a typical lung HRCT pattern and sclerosing cholangitis with sclerosis of the biliary tree on liver biopsy. The LCH diagnosis was previously performed during childhood (range: 5.4 to 15 years old) in 4 patients who were included in the study at adulthood (range: 19.5 to 37.8 years old).

**Table 1:** Characteristics of the LCH patients at the time of initiation of vinblastine

Characteristic	*N* = 35
Age, years, median, [IQR]	33 [28–42]
Male sex, n (%)	23 (66%)
Smoker, n (%)	18 (53%)
Ex-smoker	9 (27%)
Non-smoker	7 (20%)
LCH localizations, n (%)	
Bone	27 (77%)
Lung	17 (49%)
Pituitary stalk	14 (40%)
Diabetes insipidus	14 (40%)
Anterior hypophysis dysfunction^a^	9 (26%)
Skin	10 (29%)
Peripheral lymph nodes	5 (14%)
Mucosa	3 (9%)
Liver^b^	4 (11%)
CNS^c^	3 (9%)
Gut	1 (3%)
Hematologic involvement^b,d^	1 (3%)
Spleen^d^	1 (3%)
Soft tissue	1 (3%)
MS LCH	28 (80%)
RO (−)	24 (69%)
RO (+)	4 (11%)
SS bone LCH	7 (20%)
UFB with risk bone lesions	4 (11%)
MFB	3 (9%)

*IQR* interquartile range, *CNS* central nervous system, *MS* multisystem, *LCH* Langerhans cell histiocytosis, *RO* risk organ, *SS* single system, *UFB* unifocal bone, *MFB* multifocal bone

^a^All of these patients had concomitant diabetes insipidus

^b^Risk organ involvement

^c^CNS involvement was contiguous to bone lesions in one patient

^d^Present in the same patient

The median time between LCH diagnosis and vinblastine treatment was 1.4 years [IQR 0.3–4.1]. Ten (29%) patients had previously received systemic steroids. Other previous treatments included bone surgery (*n* = 7) and radiotherapy (*n* = 3; bone *n* = 2, hypophysis *n* = 1).

Three patients had received vinblastine in childhood (LCH-I protocol, *n* = 1; LCH-III, *n* = 1; and vinblastine + cyclophosphamide followed by vincristine + procarbazine, *n* = 1 15, 4 and 27 years before inclusion, respectively).

All patients had AD at the time of initiation of vinblastine. Among the 17 patients with lung involvement, 10 were smokers, 4 ex-smokers, 2 non-smokers and the smoking status was unknown in one patient. Twenty-eight patients (80%) had MS LCH, and 7 patients had SS bone disease (UFB with risk bone lesions *n* = 3, MFB *n* = 4). Four patients (11%) had RO involvement (Table 1).

### Description of treatment regimen

**Fig. 1:**
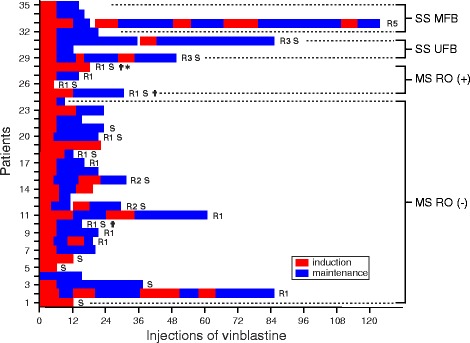
Detailed course of vinblastine received by the 35 LCH patients during the study period. Patients are classified according to the Histiocyte Society criteria: multisystem (MS) LCH without or with risk organ (RO) localizations; single system (SS) LCH distinguishing unifocal (UFB) and multifocal (MFB) bone disease. † denotes death; R reactivation/worsening followed by the number of episodes; S switched treatment; * liver transplantation

The regimen and duration of treatment received by the patients during the study period are detailed in Fig. 1. The standard regimen included at least one course of induction treatment with 6 weekly pulses of vinblastine (6 mg/m^2^, not exceeding 10 mg). In case of response after the first course of induction treatment, vinblastine was further given on a 3-week basis (maintenance treatment) for at least 6 months before 2001 (according to LCH I-II HS protocols) [15, 16] and for 12 months after 2001 (LCH-III HS protocol) [17]. For patients who did not respond after the first induction treatment, a second induction course could be tried, and if needed, the patients were switched to second-line therapy. As shown in Fig. 1, only patient #4 did not receive an initial course of induction. Six patients received vinblastine injections at a higher dose than 10 mg/day.

Prednisone was administered with vinblastine in 34 patients, with a median dose of 40 mg/m^2^ [IQR 15–40] and 60 mg/day [IQR 19–70].

Apart from hormonal substitution received by the patients with pituitary stalk involvement, three patients were receiving treatment for diabetes mellitus before inclusion in the study, and one was treated for pulmonary hypertension (oxygen, bosentan, oral anticoagulant). Trimethoprim/sulfamethoxazole was initiated at the same time as vinblastine in 11 patients (one also received valaciclovir).

### Disease response after the first course of treatment

The median duration of the first course of treatment was 7.6 months [IQR 5.9–11.5], and the median cumulative dose of vinblastine was 160 mg [IQR 120–212]. The median duration of the first course of treatment in patients treated before and after 2001 was 6.2 [IQR 5.3–12.7] and 7.8 months [IQR 6.2–11.3] (*p* = 0.79), respectively.

Twenty-five patients (71%) were responders (NAD *n* = 8; AD/better *n* = 17), 4 patients (11.5%) were intermediate responders (AD stable *n* = 1; AD mixed *n* = 3), 4 patients (11.5%) were non-responders (progressive disease), and 2 patients did not provide a clear disease state evaluation.

At the end of the first course of vinblastine treatment, 5 MS LCH patients were switched to cladribine as a second-line treatment because of progressive (*n* = 3) or intermediate mixed (*n* = 2) disease, after one or two courses of induction (patients #1,5,6) or at the end of maintenance treatment (patients #3 and #21) (Fig. 1). Cladribine was initiated for bone (*n* = 3), skin and lymph node (*n* = 1), and lung (*n* = 1) involvement.

### Response to treatment of LCH lung involvement

**Fig. 2:**
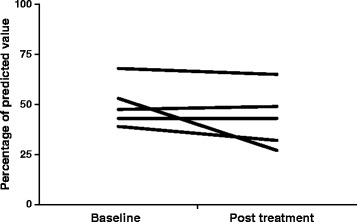
Effect of vinblastine + steroids on the forced expiratory volume in one second (FEV_1_) in the 5 LCH patients with impaired lung function at the time of initiation of treatment. Median time of treatment 7 months, [IQR 5-9]

Specific evaluations were available for 13/17 patients (76%) with lung involvement, and 8 were asymptomatic with minimal impairment of lung function. Five patients had impaired pulmonary function at the time of initiation of vinblastine. At the end of treatment (median time of 7 months, [IQR 5-9], the forced expiratory volume in 1 s (FEV_1_) worsened in 3 patients and remained stable in the 2 remaining patients under treatment (Fig. 2).

### Reactivations of disease

**Fig. 3:**
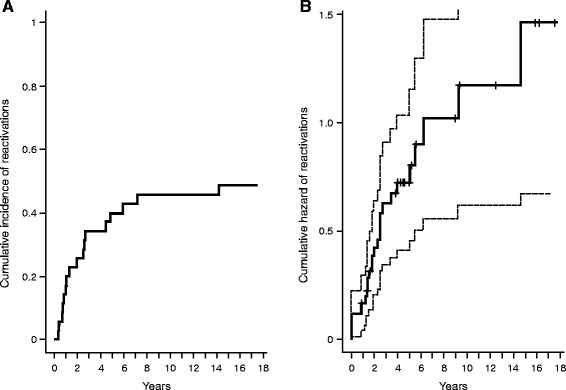
**a** Cumulative incidence of reactivation/worsening after the first course of vinblastine treatment. **b** Cumulative hazard of reactivation/worsening over the study period

The median time of follow up of the patients in the study was 83.3 months [IQR 52.1–137.3]. After the first course of vinblastine treatment, among the 25 responders, LCH recurred in 15 patients (NAD *n* = 4, AD/better *n* = 11) within a median time of 23.2 months [IQR 9.2–45.1]. Two and five of these patients experienced LCH reactivations within 6 and 12 months after the end of first course of treatment, respectively. The probability of recurrence of LCH at 6 and 12 months after the end of the first course of treatment in responders was 8% (95% CI 0–18.7%) and 20% (95% CI 4–36%), respectively. Two patients with intermediate responses (1 stable and 1 mixed) worsened within 15.2 and 171 months after the end of treatment. The cumulative incidence of LCH reactivation/worsening after the first course of vinblastine treatment is shown in Fig. 3a. At 5 years, the cumulative incidence of LCH reactivation/worsening was 40%.

Throughout the study period, 27 LCH reactivations or instances of worsening occurred (25 recurrences and 2 instances of worsening in 2 patients with intermediate stable and intermediate mixed disease, respectively). These reactivations/instances of worsening were observed in 17 patients (1 episode, *n* = 12; 2 episodes, *n* = 2; 3 episodes, *n* = 2; and 5 episodes, *n* = 1). The LCH localization and stratification (SS *vs.* MS disease) at the time of reactivation/worsening disease as well as the treatments used are detailed in Table 2. Among the 27 episodes, 13 (48%) were retreated with vinblastine in 8 patients.

**Table 2:** LCH localizations and stratification (SS *vs.* MS disease) observed during the 27 episodes of disease reactivation/worsening observed in 17 patients and associated with the treatments used during the study

Characteristic	*N* = 27
SS LCH, n (%)	11 (41%)
*Bone*	11
MS LCH, n (%)	16 (59%)
*Bone, DI and AHD, CNS*	2
*Bone, skin, oral mucosa, and DI*	1
*Bone, skin, vulvar mucosa*	2
*Bone, skin, lymph nodes*	1
*Lung and bone*	2
*Lung, bone, and DI*	1
*Lung, bone, liver, skin, oral mucosa, DI and AHD*	1
*Lung, liver*	1
*Lung, lymph nodes,*	1
*Lung, skin, DI*	2
*Skin, anal mucosa, liver*	1
*Skin, oral and anal mucosa, liver, hematologic involvement, spleen*	1
*Treatment with reactivations*	
*No treatment*	3
*Bone surgery*	1
*AINS*	1
*Vinblastine (and steroids)*	13
*Cladribine*	3
*Cladribine/MTX*	1
*Cladribine/bisphosphonates*	1
*VP16/aracytine*	1
*Steroids*	1
*IFN alpha* ^a^	1
*Liver transplantation*	1

*LCH* Langerhans cell histiocytosis, *SS* single system, *MS* multisystem, *CNS* central nervous system, *DI* diabetes insipidus, *AHD* anterior hypophysis dysfunction

^a^Administered for ECD occurrence (mixed histiocytosis)

Overall, the median duration of vinblastine treatment throughout the entire study was 9.7 months [IQR 5.7-17.1] and extended up to 70 and 98 months for two patients. The cumulative median dose of vinblastine received by the patients during the study was 205 mg [IQR 135–228.5].

At the time of the last vinblastine treatment, 25 patients (71%) were responders (NAD *n* = 12; AD/better *n* = 13). Patients were followed for a median time of 50.3 months [IQR 22.9–112.6]. LCH subsequently recurred in 10/25 (40%) patients within a median time of 28.9 months [IQR 9-31.5].

The cumulative hazard of reactivation/worsening during the study period is shown in Fig. 3b. This displays how the risk of reactivation/worsening, accounting for all the 27 observed events in the patients, increased over time. The median time of occurrence of reactivation/worsening was 48.1 months (95% CI 30.4-not reached).

There was no evidence of any factor predicting reactivation/worsening (Table 3). The risk of reactivation/worsening was not influenced by LCH localization, disease stratification (SS/MS) or treatment.

**Table 3:** Univariate analyses of the prognostic factors associated with reactivation/worsening after the first course of vinblastine

Characteristic	*N* = 35	HR (95% CI)	*p*-value
Age at vinblastine, years	33 [28–42]	1.03 (0.99; 1.08)	0.13
Male sex, n (%)	23 (66%)	0.67 (0.26; 1.75)	0.41
Smoking status			
Non-smoker	18 (53%)	1.00	
Ex-smoker	9 (27%)	1.15 (0.26; 4.95)	0.86
Smoker	7 (20%)	0.91 (0.24; 3.50)	0.89
LCH localizations, n (%)			
Bone	27 (77%)	0.56 (0.21; 1.48)	0.27
Lung	17 (49%)	0.56 (0.21; 1.48)	0.24
Diabetes insipidus	14 (40%)	0.60 (0.22; 1.64)	0.31
Anterior hypophysis dysfunction	14 (40%)	0.51 (0.15; 1.79)	0.29
Skin	9 (26%)	1.95 (0.74; 5.14)	0.18
Peripheral lymph nodes	10 (29%)	0.67 (0.15; 2.95)	0.59
Classification of LCH			
MS	28 (80%)	1.00	
SS		1.46 (0.41; 5.23)	0.56
First vinblastine dose^a^		1.00 (0.96; 1.05)	0.86
Duration of first course, months		0.97 (0.89; 1.05)	0.41

*HR* hazard ratio, *CI* confidence interval, *LCH* Langerhans cell histiocytosis, *MS* multisystem, *SS* single system

^a^Reported HR represents the increased risk of reactivation/worsening for two patients exhibiting a difference of 10 mg in vinblastine dose

### Toxicity

A total of 27 side effects were observed in 16 (46%) patients treated with vinblastine + steroids. The adverse events and their grading are detailed in Table IV. Neutropenia (grade 3/4) was observed in 6 of these patients, although none developed infection. Two patients developed grade 3 steroid-induced side effects (Table 4). These two patients had pan hypopituitarism, which might have favored the weight gain.

**Table 4:** Adverse events observed under vinblastine + steroid treatment throughout the study

Characteristic	*N* = 35
At least one adverse event, n (%)	16 (46%)
Non-neurological adverse events	14 (40%)
Neutropenia	6 (17%)
grade 3/4	4/2
Herpes zoster	1 (3%)
Catheter-related septicemia (grade 4)	1 (3%)
Digestive disorders	4 (11%)
grade 1	3
grade 2^a^	1
Alopecia (grade 1)	1 (3%)
Muscular pain (grade 1)	2 (6%)
Steroid related adverse events	2 (6%)
weight gain (grade 3)	1
weight gain and de novo diabetes (grade 3)	1
Neurological adverse events	9 (26%)
Peripheral sensitive neuropathy (grade 2)	9 (26%)
Peripheral motor neuropathy^b^ (grade 2)	1 (3%)

^a^Transient paralytic ileus

^b^Concomitant to sensitive neuropathy

Details on the risk factors for the development of peripheral neuropathy were available for 34 patients. Eight patients had risk factors of neuropathy before vinblastine: liver disease *n* = 2; thalidomide *n* = 1; diabetes *n* = 4; stroke *n* = 1; peripheral nerve compression *n* = 1 (right ulnar and left peroneal). The patient who was previously treated with thalidomide also had diabetes mellitus.

Nine patients developed grade 2 neurological toxicity (peripheral sensitive neuropathy in all patients, associated with decreased strength of the right hand in one patient) after a median time of 2 months [IQR 1.2–15.9] and at a median cumulative dose of 70 mg [IQR, 55–77] vinblastine. Vinblastine had to be interrupted in only 1 patient who developed peripheral sensitive neuropathy after 5 injections (cumulative dose 50 mg), and sequelae was not observed after treatment interruption. For the other patient, vinblastine was transiently interrupted for 1 month, and he ultimately received a cumulative dose of 168 mg and did not show the recurrence of neuropathy or sequelae. Only one patient had peripheral sensitive neuropathy sequelae related to previous treatment with thalidomide.

No risk factors were identified as associated with neurological toxicity in the univariate analyses (Table 5).

**Table 5:** Univariate analyses of risk factors for neurotoxicity^a^

Characteristic	No neurologic toxicity (=25)	Neurologic toxicity (*n* = 9)	*p*-value
Age, years, median, [IQR]	31.6 [28–39.6]	33.6 [25.7–48.7]	0.74
Male sex, n (%)	17 (71%)	5 (56%)	0.44
Smoker, n (%)	12 (52%)	6 (67%)	0.28
Ex-smoker	5 (22%)	3 (33%)	
Non-smoker	6 (26%)	0	
LCH stratification			
MS	20 (80%)	7 (78%)	1.00
SS	5 (21%)	2 (22%)	
Previous risk factor of neuropathy^a^	5 (20%)	3 (33%)	0.65
Vinblastine Treatment			
Number of injections, median, [IQR]	17 [13.5; 21]	29 [21; 49]	0.085
Cumulative dose, mg, median, [IQR]	152.8 [120; 210]	168 [130; 220]	0.38
Duration of exposure, months, median, [IQR]	7.6 [5.4; 12.0]	7.6 [6.2; 9.9]	0.90

*IQR* interquartile range, *LCH* Langerhans cell histiocytosis, *MS* multisystem, *SS* single system

^a^Data on risk factors were available for 34 patients

### Permanent consequences

At the final vinblastine treatment, permanent consequences of LCH were present in 15 patients (43%), with pituitary involvement in 14 patients (40%, diabetes insipidus *n* = 14, associated anterior hypophysis dysfunction *n* = 9); respiratory impairment in 4 patients (11.5%); sclerosing cholangitis in 1 patient (3%); and central nervous system (CNS) impairment in 1 patient (3%). All these permanent consequences were present at the time of vinblastine initiation.

### Survival

Three patients died at the end of the study. One patient died 6 months after liver transplantation, and another died of meningeal hemorrhage secondary to thrombocytopenia induced by interferon alpha given for mixed LCH/ECD 41 months after initiation of vinblastine. The latter patient, without RO involvement, developed a secondary acute myeloid leukemia 93 months after the initiation of treatment. This patient was heavily treated (vinblastine + cyclophosphamide followed by vincristine + procarbazine) for LCH during his childhood.

**Fig. 4:**
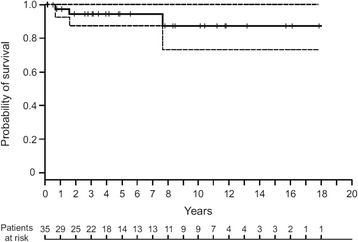
Kaplan-Meier survival estimates of the 35 LCH patients during the study period. Hash marks indicate censored patients. Dashed lines indicate the limits of the 95% confidence interval

The Kaplan-Meier survival curve is shown in Fig. 4. The 10-year survival rate was 86.2% (95 CI, 71.8–100%). The 2 patients who died because of their LCH had RO localizations (liver involvement and “Letterer-Siwe” followed by ECD). Death related to LCH was not observed in patients without RO involvement.

## Discussion

In this multicenter study, we found the following salient results: 1) except for lung involvement, a vinblastine + steroids regimen was an efficient first-line treatment for adult LCH patients; 2) this treatment was well tolerated; 3) LCH had a propensity to recur over time, mostly later than one year after treatment completion; and 4) the presence of RO involvement was associated with worse prognoses and accounted for LCH-related deaths.

Although this multicenter study was not a prospective trial, the therapeutic regimen of vinblastine + steroids administered to the patients was in accordance with protocols designed by the HS for pediatric LCH [15, 17]. The median duration of the first course of vinblastine was 7.6 months, i.e., half way between the durations evaluated in LCH-I and LCH-III HS trials [15, 17]. The rate of disease response (NAD and AD/better) of 71% after the first course of vinblastine was even slightly higher than in the LCH-I study [15], which was probably because RO involvement occurs much less often in adult patients as was the case in our series.

The few data available concerning vinblastine treatment in adult LCH patients mainly concern patients with bone disease [11, 21–23]. Individual case reports showed a rather good response to vinblastine [21–23]. In contrast, in a series of adult patients with bone disease, [11] found that 84% patients treated with vinblastine had a poor response, defined by either the absence of response or relapse of LCH within a year. These criteria for disease response are different from those used in LCH HS trials [15, 17]. However, even using the same criteria, vinblastine was clearly more effective in our study. Notably, LCH bone involvement was by far the most frequent localization (77%) in our patients. Furthermore, no difference in response to treatment was observed for the 20% patients with SS bone LCH, and no association between LCH localizations and outcome was demonstrated.

Apart from scant case reports [24, 25], the present study provides the first assessment of the effects of vinblastine as a first-line treatment in a series of adults with MS-LCH. The international registry of the HS on adult LCH comprised patients with MS LCH treated with vinblastine, but no data were provided on the disease response to this treatment [26]. Here, we found that vinblastine was an effective treatment for these patients except for those with progressive lung involvement.

There are virtually no data on the efficiency of vinblastine in LCH patients with lung involvement. We could identify one pediatric case in which vinblastine had no effect on impaired lung function [27]. Here, we found that vinblastine + steroids had either no effect or did not preclude further lung function deterioration in patients with lung involvement. For these patients, cladribine is a promising treatment [28–30].

A considerable problem associated with LCH is its propensity to recur [8, 9]. Thus, the LCH-III HS trial tested the prolongation of vinblastine treatment to 12 months in patients without RO involvement [17]. The 40% probability of LCH recurrence at 5 years found in our study is similar to the 37% 5-year probability of recurrence observed in the 12-month arm in RO-negative patients in the LCH-III study [17]. We also found that only 20% of these recurrences occurred within the year following the end of the first course of vinblastine. Some patients experienced several reactivation episodes during the study, at a median time of occurrence of 48 months. Half of the observed reactivations were treated again with vinblastine. Considering the whole study population, 70% of the patients were responders at the end of last course of vinblastine, approximately half were NAD and half were AD/better.

Forty percent of all reactivations presented as SS bone disease, and 60% presented as MS LCH. We did not find any association between LCH localization or stratification (SS *vs.* MS disease) and reactivation of the disease. However, the small number of patients may have limited the statistical power to detect such a finding. Additional studies are needed to evaluate the best therapeutic strategies to reduce the rate of long-term LCH recurrences.

An important finding of our study was the overall good tolerance of vinblastine + steroid treatment. Grade 3/4 adverse events occurred in 9 (26%) patients and were related to steroids in two of the patients. Although peripheral sensitive neuropathy was observed in 26% of the study population, it was of grade 2 in all cases. These neurological adverse events did occur early in the course of vinblastine treatment. Notably, the treatment had to be definitively interrupted in only one patient without sequelae. Our results are discordant with those of Cantu *et al*., who reported grade 3–4 toxicity in 75% of their adult patients treated with vinblastine [11]. In this regard, vinblastine was reported to be well-tolerated in all but one previously reported case [22–25]. In a study evaluating the effects of vinblastine on CNS-LCH, 7 adults were included, and only one of them presented a mild peripheral neuropathy [31]. Our results are strengthened by the multicenter design of our study.

Forty percent of the patients had permanent consequences by the end of the study. These sequelae involved the pituitary stalk in all but one patient, which is similar to the incidence reported in pediatric studies [15, 20]. Because of lung involvement in nearly half of our patients and the lack of effectiveness of vinblastine on lung function, respiratory impairment was the second most frequent permanent consequence observed. All of these permanent consequences were present at the time of initiation of vinblastine.

The 10-year survival rate of our patients was 86%, and 3 patients died during the study period. Two of these patients had RO involvement, which confirms the pejorative prognosis of these LCH localizations in adults, as in childhood LCH [9, 17, 20]. The last patient without RO involvement had been heavily treated with chemotherapy in his childhood and ultimately developed fatal secondary acute myeloid leukemia 7.5 years after vinblastine treatment administered in his adulthood, a well described risk in LCH [32]. When excluding these 3 patients, no death was observed in the study period, which is in accordance with results of both the LCH-III study and the adult international HS registry in RO negative patients [17, 26].

Our study has several limitations. Because of its retrospective nature, patients were not evaluated at the same time. Similarly, although it is the largest series reported, the small number of patients could have made it difficult for us to detect differences in response in subcategories of patients.

## Conclusions

In this multicenter study, we showed that vinblastine is an effective and well-tolerated first-line chemotherapy for adult LCH patients except for patients with progressive lung involvement. HH reactivation during long-term follow up. As in pediatric LCH, the presence of risk organ involvement has a negative impact on patient prognosis.

## Abbreviations

AD: Active disease
AHD: Anterior hypophysis dysfunction
CI: Confidence interval
CNS: Central nervous system
CTCAE: Common terminology criteria for adverse events
DI: Diabetes insipidus
ECD: Erdheim-chester disease
FEV_1_: Forced expiratory volume in 1 s
HR: Hazard ratio
HRCT: High-resolution computed tomography
HS: Histiocyte society
IQR: Interquartile range
LCH: Langerhans cell histiocytosis
MFB: Multifocal bone
MS: Multisystem
NAD: Non active disease
NCI: National Cancer Institute
RO: Risk organs
SS: Single system
UFB: Unifocal bone
